# Stroke associated with primary membranous nephropathy in a young adult: Case report

**DOI:** 10.7705/biomedica.7117

**Published:** 2024-05-31

**Authors:** Juan Pablo Morales

**Affiliations:** 1 Departamento de Medicina Interna, Universidad del Valle, Cali, Colombia Departamento de Medicina Interna Universidad del Valle Cali Colombia; 2 Hospital Universitario del Valle Evaristo García, Cali, Colombia Hospital Universitario del Valle Evaristo García Cali Colombia

**Keywords:** Cerebral infarction, stroke, young adult, nephrotic syndrome, glomerulonephritis, membranous., infarto cerebral, accidente cerebrovascular, adulto joven, síndrome nefrótico, glomerulonefritis membranosa.

## Abstract

**Introduction.:**

Stroke in young individuals is becoming increasingly prevalent worldwide. Its causes can vary widely, so a thorough investigation by a multidisciplinary team is needed. Pinpointing the precise underlying pathology responsible for the stroke yields benefits for patients, particularly in recurrent events.

**Case presentation.:**

A 38-year-old man presented to the emergency department with symptoms suggestive of stroke, including right hemiparesis, dysarthria, ataxic gait, and right central facial palsy. The brain magnetic resonance image revealed an ischemic lesion located in the left basal ganglia and near the corona radiata. Following an extensive workup, a diagnosis of nephrotic was reached. Histopathology and the exclusion of secondary causes confirmed primary membranous nephropathy as the underlying condition.

The patient underwent treatment tailored to address the specific glomerulopathy, along with anticoagulation therapy and immunosuppression as per current guidelines. Subsequent assessments showed stabilization of renal function, resolution of the edema, and the absence of new thromboembolic events during follow-up.

**Conclusion.:**

The nephrotic syndrome should be recognized as a potential underlying cause of stroke in young patients and, therefore, it should be included in the differential diagnosis during the evaluation of patients with coagulopathies. Nephrotic syndrome screening may be done by conducting a simple urinalysis readily available in most healthcare facilities. This underlines the importance of considering renal pathology in the assessment of stroke etiologies, especially when coagulation abnormalities are present.

## Resumen

**Introducción.:**

Los eventos cerebrovasculares en los jóvenes son un problema creciente en todo el mundo. Su etiología puede ser variada y requieren un trabajo riguroso de un equipo multidisciplinario. La identificación de la enfermedad específica que conduce al ictus tiene un impacto beneficioso en los pacientes, especialmente en aquellos con eventos recurrentes.

**Presentación del caso.:**

Se presenta el caso de un hombre de 38 años que acudió al servicio de urgencias con hemiparesia derecha, disartria, ataxia y parálisis facial central derecha. La resonancia magnética cerebral reveló una lesión isquémica localizada en los ganglios basales izquierdos, cerca de la corona radiada. Después de un estudio exhaustivo, se estableció el diagnóstico de síndrome nefrótico. No obstante, al analizar las características histopatológicas y descartar otras causas secundarias, el diagnóstico final fue una nefropatía membranosa primaria. El paciente recibió tratamiento específico para su glomerulopatía, anticoagulación e inmunosupresión según las guías vigentes.

Durante el seguimiento, se encontró estabilización de la función renal, el edema se resolvió y no se identificó ningún nuevo evento tromboembólico.

**Conclusión.:**

El síndrome nefrótico debe considerarse entre las posibles causas del ictus en pacientes jóvenes y debería tenerse en cuenta en los estudios de coagulopatías. El tamizaje de esta enfermedad requiere únicamente un uroanálisis, el cual está disponible en la mayoría de los centros de atención de salud.

Ischemic stroke in young adults is increasingly recognized as a significant global health issue, affecting both developed and developing nations. Its impact on healthcare systems is a major concern due to the resulting disability in young people in their productive working years ^1^. The definition of “young adult” often varies according to different studies, but it generally includes ages from 18 to 50 years. Recently, there has been a rise in the incidence of ischemic stroke among young adults despite some regions lacking comprehensive reporting. Currently, approximately one in ten ischemic strokes occurs in this demographic group ^2^.

While chronic diseases such as high blood pressure, diabetes, and obesity are on the rise among young people, the diagnosis of ischemic stroke in this population should prompt physicians to investigate less common etiologies including vascular, cardiac, hematologic, autoimmune, toxic, and genetic conditions ^3^. Traditionally, the Trial of Org 10172 in Acute Stroke Treatment (TOAST) has been utilized to study, categorize, and report stroke etiology. Etiology is classified as “probable” when the results align with the patient's clinical presentation and imaging findings, and as “possible” when conclusive evidence is lacking or relevant investigations are not performed ^4^. However, ischemic stroke in young adults warrants heightened attention and more extensive diagnostic evaluation to rule out additional conditions, since treating the underlying condition can significantly impact the recurrence of stroke events and improve prognosis. This case report illustrates how timely identification of the underlying condition can positively influence patient outcomes.

## Case presentation

A 38-year-old male patient arrived at the emergency department complaining of a 24-hour right hemiparesis, dysarthria, and ataxic gait. He denied similar previous episodes, seizures, chest pain, or loss of consciousness. In the last nine months, he had presented ankle edema and mild dyspnea on exertion. He was medicated with hydrochlorothiazide (50 mg) plus amiloride (5 mg) once daily, furosemide 40 mg daily, losartan 50 mg twice daily, nifedipine 30 mg, and atorvastatin 40 mg once daily to control elevated blood pressure and dyslipidemia.

In the physical examination on admission, his vital signs were: blood pressure, 162/100 mm Hg, heart rate 99 beats per minute, respiratory rate 17 per minute, temperature 36.1 °C, weight 69 kg, and height 1,7 meters [body mass index (BMI): 23,8 kg/m^2^]. Neurological abnormalities included right central facial nerve palsy and a tendency to fall to the right side during the Romberg test. Sensitivity and musculoskeletal reflexes were normal. There was mild bilateral ankle edema with no ascites or jugular vein engorgement. Initial tests showed abnormal brain computed tomography (CT), and blood tests indicated abnormal kidney function, subclinical hypothyroidism, and mild normocytic anemia (table 1).

**Table 1:** Laboratory tests at admission and one month after discharge

Test	Admission	One month later	Normal range
Serum creatinine (mg/dl)	1.57	1.4	0.7-1.2
Blood urea nitrogen (mg/dl)	37.5	43	9.0-20
Potassium (mEq/L)	4.6	-	3.5-5.1
Sodium (mEq/L)	135	-	137-145
Total calcium (mg/dl)	7.49	-	8.4-10.2
Blood glucose (mg/dl)	94	-	74-106
Thyroid stimulating hormone (μIU/ml)	8.53	4.54	0.47-4.68
Thyroxine (μIU/ml)	1.12	-	0.78-2.19
Aspartate aminotransferase (IU/L)	19	-	17-59
Alanine aminotransferase (IU/L)	8	-	0-50
White blood cells (1 x 103/μl)	11.21	-	4.05-11.84
Hemoglobin g/dl	10.9	-	13.5-17.2
Mean corpuscular volume (fl)	89.2	-	80-99
Platelets (1 x 103/μl)	408	-	150-450
Red cell distribution width (%)	12.9	-	11.5-14.7
HIV	Negative	-	-
Hepatitis B antibodies	Negative	-	-
Hepatitis C antibodies	Negative	-	-
Syphilis	Negative	-	-
A1C glycated hemoglobin (%)	4.78	-	0-6.5
Antinuclear antibodies	Negative	-	-
Antiphospholipid antibodies	Negative	-	-
Albumin (g/dl)	1.6	1.4	3.5-5
Total cholesterol (mg/dl)	310	365	0-200
High density lipoproteins (mg/dl)	43	66	40-60
Low density lipoproteins (mg/dl)	190	187	< 100
Triglycerides (mg/dl)	1,575	408	0-150
Antithyroid antibodies	Negative	-	-
Urine protein-creatinine ratio (mg/mg)	9.82	9.47	

**Figure 1:**
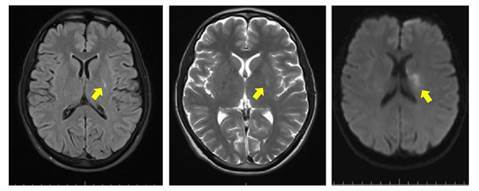
Brain magnetic resonance imaging: left: T1 sequence; middle: T2 sequence; right: diffusion sequence. White arrows show a small lesion (2.5 cm in size) with restriction to diffusion, compromising the left basal ganglia region, the caudate nucleus, and part of the corona radiata.

Ischemic stroke in a young adult was the initial diagnostic hypothesis. Several additional tests were performed to clarify the TOAST classification, including transesophageal echocardiogram, neck and brain computed tomography angiography, and 24-hour Holter electrocardiogram. However, only brain magnetic resonance imaging showed an ischemic lesion in the left basal ganglia near the corona radiata (figure 1). Other test results were within normal limits. Additional blood tests included rheumatologic tests, screening for infectious diseases, lipid panel, complementary kidney function tests, and serum albumin. Laboratory results showed massive 24 hour-proteinuria (10 g), severe dyslipidemia, and a marked decrease in serum albumin. The estimated glomerular filtration rate was 57 ml/min/1.73 m^2^ (using the equation of the Chronic Kidney Disease Epidemiology Collaboration - CKD-EPI). With these results, nephrotic syndrome criteria were met. Initial management for glomerular disease included renin-angiotensin axis inhibitors, blood pressure control, sodium and protein intake restriction, and lipid reduction with high- intensity statins.

**Figure 2:**
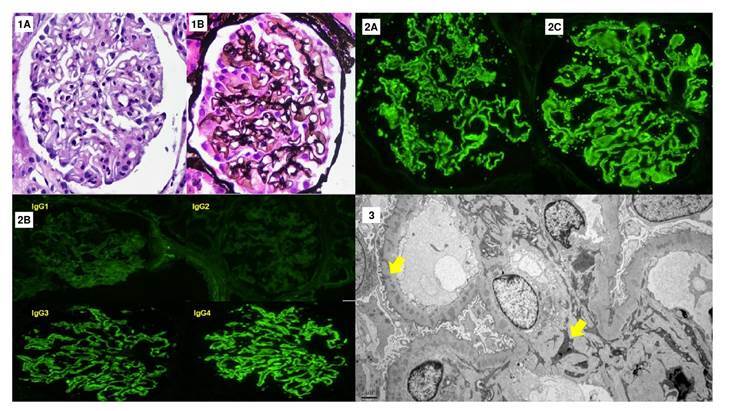
Renal biopsy. Twenty glomeruli, one sclerosed, are observed. **1A.** The glomeruli show marked podocyte activation associated with diffuse thickening of the glomerular basement membranes in the hematoxylin-eosin staining (40X). **1B.** Evidence of spikes and holes in the methenamine silver stain (40X). The interstitium shows few areas of interstitial fibrosis and tubular atrophy. **2A-B.** Direct immunofluorescence stains: Intense granular staining is observed in the basement membranes with IgG (4+), C3 (2+), kappa (4+), lambda (4+) (40X), and anti-PLA2R (2-3+). 2C. IgG subclasses show a predominance of IgG4 (4+) over IgG3 (3+), IgG2 (1+), and IgG1 (2+). **3.** Electron microscopy showing electron-dense deposits of mesangial and intramembranous locations (arrows), some with peaks (5,000X

Due to alterations in kidney function tests and the possibility of a serious complication like stroke, the nephrotic syndrome was considered severe. We proposed a kidney biopsy suspecting probable primary glomerulopathy with the possibility of starting immunosuppression therapy. The diagnosis of primary membranous nephropathy was established after analyzing the pathology specimen through basic stains, immunohistochemistry, and electron microscopy (figure 2). Serum anti- anti-phospholipase A2 receptor (PLA2R) antibodies or other membranous nephropathy-associated antibodies). Anti-PLA2R antibodies in serum or other membranous nephropathy-associated antibodies were not measured. Due to low serum albumin levels and massive proteinuria, full anticoagulation therapy was started, initially with low molecular weight heparin and then warfarin.

According to the latest improving global outcomes (KDIGO) guideline on glomerular disease, and its severity in this patient, immunosuppressive therapy was started in a six-month scheme including cyclophosphamide at a dose of 750 mg/m^2^ during months two, four, and six, and methylprednisolone at a dose of 1 g during months one, three and five, i.e., the so-called Ponticelli modified scheme. The immunosuppressive therapy was administered along with trimethoprim-sulfamethoxazole at a dose of 800/160 mg for *Pneumocystis jirovecii* prophylaxis every other day.

One month after, new tests were taken during the outpatient clinic follow-up. There was a significant decrease in lipid panel values and serum albumin, and kidney function remained stable; no new ischemic events were documented. The ankle edema disappeared, as did the dyspnea with exertion.

### Ethical standard

The author confirms having read the journal’s position on ethical publication and states that this report is consistent with those guidelines. Written informed consent was obtained from the patient and a witness to publish the case and accompanying images or laboratory results.

## Discussion

Nephrotic syndrome, particularly membranous nephropathy, is a complex and multifactorial hypercoagulable state. It arises from high urine protein excretion and significant hypoalbuminemia, which indirectly leads to a reduction of natural anticoagulant proteins such as C and S proteins, as well as antithrombin III ^5^. Traditionally, nephrotic syndrome has been associated with venous thromboembolic events, whose overall incidence among patients with nephrotic syndrome is around 25%. In the subgroup of membranous nephropathy patients, renal vein thrombosis can occur in up to 37% ^5^. Arterial thrombotic events may also occur in rare cases, although most of the evidence available comes from case reports. Current understanding of the mechanisms and pathophysiology involved remains limited. Observational studies have provided data suggesting that platelet function may play a role in the development of arterial thrombosis observed in nephrotic syndrome. However, the precise alterations are not yet fully understood ^6^.

In this case of ischemic stroke in a young man, after the TOAST strategy, extensive studies were conducted to rule out secondary causes. Ultimately, the diagnosis of primary membranous nephropathy was confirmed through histopathology and immunofluorescence features. A significant role was attributed to anti-PLA2R antibodies that confirmed, with high sensitivity and specificity, the diagnosis of primary membranous nephropathy subtype ^7^. In recent years, the significance of anti-PLA2R in MN has increased. Many cases of membranous nephropathy have been found to have anti-PLA2R antibodies and, in turn, these antibodies in serum have helped in diagnosing primary membranous nephropathy, thus eliminating the need for kidney biopsy. Furthermore, anti-PLA2R titers have facilitated patient follow-up and prognosis assessment ^8^^,^^9^.

The two most frequent complications of nephrotic syndrome are infections and thrombotic events. While venous thromboembolic events have been extensively described, arterial thrombosis is a highly uncommon complication ^5^. When arterial events occur, the most frequent locations are the femoral and iliac arteries, as reported by Fahal *et al.*^10^. However, this data becomes controversial in specific membranous nephropathy patient groups since the selected group consists only of membranous nephropathy patients.

In such scenarios, the most common locations for arterial thrombosis vary, with a higher frequency observed in the coronary circulation, central nervous system, and peripheral arteries. These events predominantly occur during the first year of the disease when hypoalbuminemia and proteinuria are at their peak ^11^. In a study by Roy *et al.,* there were strikingly few M membranous nephropathy patients among those with nephrotic syndrome and stroke and none in Latin America, probably due to underdiagnosis ^12^.

Once the diagnosis of membranous nephropathy-associated stroke has been established, the next challenge was to determine the treatment strategy. The KDIGO guideline recommends implementing general measures for membranous nephropathy patients, emphasizing the renin-angiotensin axis inhibitors, blood pressure control, and dietary recommendations regarding sodium and protein intake. Additionally, it is crucial to determine anticoagulation therapy for these patients. In general, patients with serum albumin levels below 2.5 g/dl would benefit from anticoagulation, preferably with heparin and warfarin, if there are no contraindications to initiating the therapy. Furthermore, recent evidence suggests that membranous nephropathy patients may benefit from antiplatelet therapy with aspirin to prevent thromboembolic events if their serum albumin is below 3.2 g/dl ^6^.

Another challenge is the initiation of immunosuppressive therapy in membranous nephropathy subjects as it depends on the patient’s risk level. In this case, the risk was high to very high due to the deterioration of his glomerular filtration rate, high urine protein excretion, and the occurrence of the stroke, a major vascular complication. Considering the data and available resources, an immunosuppressive regimen of oral cyclophosphamide and steroids was initiated following the modified Ponticelli protocol ^13^. In other contexts, a rituximab-based protocol may be chosen ^14^, but in the present case, it had limited availability, and the complete treatment could not be guaranteed. This aspect must be considered for long-term immunosuppressive protocols, as getting oral cyclophosphamide can be difficult in some Latin American countries. Interestingly, the study by Luzardo *et al*. showed acceptable outcomes in terms of efficacy and safety when oral cyclophosphamide was switched to the parenteral route ^15^. However, this new information should be taken with caution, given the retrospective nature of the data.

## Conclusions

Stroke in young individuals is a major cause of disability. Identifying the underlying etiology of stroke should be prioritized to reduce the risk of recurrent thromboembolic events. It is crucial to maintain a low suspicion threshold in many low-incidence pathologies and follow systematic strategies such as TOAST. As in this case, certain conditions, including nephrotic syndrome, may not be included in most protocols. In the case of young patients with stroke and edema, nephrotic syndrome should be considered using urinalysis as an initial screening test followed by further evaluation.
